# Sir Francis Bacon’s Cipher Story

**Published:** 1895-05

**Authors:** 


SIR FRANCIS BACON'S CIPHER STORY. Discovered and deciphered by Orville W. Owen, M. D. Book IV. Detroit and New York: Howard Publishing Co., 1894. Paper cover. Price, 50 cents.

The readers of this journal have, from time to time, been informed of the progress of this astounding literary work by reviews of the different books as they have been issued from the press. For the convenience of those who wish to refresh their memories, we note that the review of the first volume was published in the number for June, 1894, at page 186, with editorial comment at page 163. The review of the second and third volumes can be found in the number for September, 1894, at page 290.

The present volume is more astonishing still. It contains the tragic history of the execution of the beautiful Mary, Queen of Scots, related in the form of a play! A play concealed by means of a cipher within a play! Could ingenuity go farther?

This play of "Queen Mary" seems to have been extracted from "Hamlet," as a number of familiar passages from that master-piece of literature occur at different places.

Bacon relates that when he first wrote "Hamlet," his mother, Queen Elizabeth, discovered it, "and then," he says, "I was lost." He was betrayed to the Queen by his father, the Earl of Leicester, who sneaked into a room adjoining one where Bacon was rehearsing the actors for his tragedy, and overheard all that passed. Leicester then reported to the Queen, who summoned Bacon before her, and roundly scolded him for exposing to the world the crimes of monarchs. Bacon protested and explained, and the Queen pretended to be convinced and reconciled. Having thrown the young author off his guard, she expressed an interest in his work, and desired to peruse it. Imprudently, the son brought the manuscript to her, and so intense was her desire to destroy it that she could hardly wait until he returned with it. The instant he entered the room, bearing the precious manuscript, she sprang at him like a tigress, wrenched the document from his hands and in an instant had torn it to pieces, without any pretense at examination, and cast it into the fire. This compelled him to rewrite it, which he did, weaving into it the cipher with which all his works abound.

According to the play the Queen was not the author of the death of Mary, though she seemed ardently to desire it. The execution was brought about by a conspiracy between Lord Burleigh, the Chancellor of England, and the Earl of Leicester, unacknowledged husband of Queen Elizabeth, and father of Sir Francis Bacon. This precious pair prepared a warrant of execution and then overawed the Queen's private secretary into forging Elizabeth's signature to the document and affixing to it the great seal of England. With this weapon in their hands, the public executioner was summoned, and so the great tragedy was accomplished.

All this the reader would best peruse for himself as the situations are exceedingly interesting and startling.

The Howard Publishing Csmpany have now finished volume V, also a play, depicting the tragedy of the Earl of Essex. This last has not been worked out by Dr. Owen, but by his clerks to whom he taught the cipher, and who, by this means were enabled to continue the work of the great decipherer himself. A review of this new volume will appear in these pages in a later issue.

